# Correction to 153 abstracts to correct abstract citation ID in the PDF version

**DOI:** 10.1093/jcag/gwag026

**Published:** 2026-09-17

Upon original publication, the abstract citation IDs in the PDF version of the abstracts listed below were incorrect.

Additionally, the abstract citation ID was omitted from the PDF version of abstract https://doi.org/10.1093/jcag/gwaf042.221.

The Publisher apologizes for these errors, which have now been corrected.

The affected abstracts are as follows:

https://doi.org/10.1093/jcag/gwaf042.185

https://doi.org/10.1093/jcag/gwaf042.186

https://doi.org/10.1093/jcag/gwaf042.187

https://doi.org/10.1093/jcag/gwaf042.188

https://doi.org/10.1093/jcag/gwaf042.189

https://doi.org/10.1093/jcag/gwaf042.190

https://doi.org/10.1093/jcag/gwaf042.191

https://doi.org/10.1093/jcag/gwaf042.192

https://doi.org/10.1093/jcag/gwaf042.193

https://doi.org/10.1093/jcag/gwaf042.194

https://doi.org/10.1093/jcag/gwaf042.195

https://doi.org/10.1093/jcag/gwaf042.196

https://doi.org/10.1093/jcag/gwaf042.197

https://doi.org/10.1093/jcag/gwaf042.198

https://doi.org/10.1093/jcag/gwaf042.199

https://doi.org/10.1093/jcag/gwaf042.200

https://doi.org/10.1093/jcag/gwaf042.201

https://doi.org/10.1093/jcag/gwaf042.202

https://doi.org/10.1093/jcag/gwaf042.203

https://doi.org/10.1093/jcag/gwaf042.204

https://doi.org/10.1093/jcag/gwaf042.205

https://doi.org/10.1093/jcag/gwaf042.206

https://doi.org/10.1093/jcag/gwaf042.207

https://doi.org/10.1093/jcag/gwaf042.208

https://doi.org/10.1093/jcag/gwaf042.209

https://doi.org/10.1093/jcag/gwaf042.210

https://doi.org/10.1093/jcag/gwaf042.211

https://doi.org/10.1093/jcag/gwaf042.212

https://doi.org/10.1093/jcag/gwaf042.213

https://doi.org/10.1093/jcag/gwaf042.214

https://doi.org/10.1093/jcag/gwaf042.215

https://doi.org/10.1093/jcag/gwaf042.216

https://doi.org/10.1093/jcag/gwaf042.217

https://doi.org/10.1093/jcag/gwaf042.218

https://doi.org/10.1093/jcag/gwaf042.219

https://doi.org/10.1093/jcag/gwaf042.220

https://doi.org/10.1093/jcag/gwaf042.222

https://doi.org/10.1093/jcag/gwaf042.223

https://doi.org/10.1093/jcag/gwaf042.224

https://doi.org/10.1093/jcag/gwaf042.225

https://doi.org/10.1093/jcag/gwaf042.226

https://doi.org/10.1093/jcag/gwaf042.227

https://doi.org/10.1093/jcag/gwaf042.228

https://doi.org/10.1093/jcag/gwaf042.229

https://doi.org/10.1093/jcag/gwaf042.230

https://doi.org/10.1093/jcag/gwaf042.231

https://doi.org/10.1093/jcag/gwaf042.232

https://doi.org/10.1093/jcag/gwaf042.233

https://doi.org/10.1093/jcag/gwaf042.234

https://doi.org/10.1093/jcag/gwaf042.235

https://doi.org/10.1093/jcag/gwaf042.236

https://doi.org/10.1093/jcag/gwaf042.237

https://doi.org/10.1093/jcag/gwaf042.238

https://doi.org/10.1093/jcag/gwaf042.239

https://doi.org/10.1093/jcag/gwaf042.240

https://doi.org/10.1093/jcag/gwaf042.241

https://doi.org/10.1093/jcag/gwaf042.242

https://doi.org/10.1093/jcag/gwaf042.243

https://doi.org/10.1093/jcag/gwaf042.244

https://doi.org/10.1093/jcag/gwaf042.245

https://doi.org/10.1093/jcag/gwaf042.246

https://doi.org/10.1093/jcag/gwaf042.247

https://doi.org/10.1093/jcag/gwaf042.248

https://doi.org/10.1093/jcag/gwaf042.249

https://doi.org/10.1093/jcag/gwaf042.250

https://doi.org/10.1093/jcag/gwaf042.251

https://doi.org/10.1093/jcag/gwaf042.252

https://doi.org/10.1093/jcag/gwaf042.253

https://doi.org/10.1093/jcag/gwaf042.254

https://doi.org/10.1093/jcag/gwaf042.255

https://doi.org/10.1093/jcag/gwaf042.256

https://doi.org/10.1093/jcag/gwaf042.257

https://doi.org/10.1093/jcag/gwaf042.258

https://doi.org/10.1093/jcag/gwaf042.259

https://doi.org/10.1093/jcag/gwaf042.260

https://doi.org/10.1093/jcag/gwaf042.261

https://doi.org/10.1093/jcag/gwaf042.262

https://doi.org/10.1093/jcag/gwaf042.263

https://doi.org/10.1093/jcag/gwaf042.264

https://doi.org/10.1093/jcag/gwaf042.265

https://doi.org/10.1093/jcag/gwaf042.266

https://doi.org/10.1093/jcag/gwaf042.267

https://doi.org/10.1093/jcag/gwaf042.268

https://doi.org/10.1093/jcag/gwaf042.269

https://doi.org/10.1093/jcag/gwaf042.270

https://doi.org/10.1093/jcag/gwaf042.271

https://doi.org/10.1093/jcag/gwaf042.272

https://doi.org/10.1093/jcag/gwaf042.273

https://doi.org/10.1093/jcag/gwaf042.274

https://doi.org/10.1093/jcag/gwaf042.275

https://doi.org/10.1093/jcag/gwaf042.276

https://doi.org/10.1093/jcag/gwaf042.277

https://doi.org/10.1093/jcag/gwaf042.278

https://doi.org/10.1093/jcag/gwaf042.279

https://doi.org/10.1093/jcag/gwaf042.280

https://doi.org/10.1093/jcag/gwaf042.281

https://doi.org/10.1093/jcag/gwaf042.282

https://doi.org/10.1093/jcag/gwaf042.283

https://doi.org/10.1093/jcag/gwaf042.284

https://doi.org/10.1093/jcag/gwaf042.285

https://doi.org/10.1093/jcag/gwaf042.286

https://doi.org/10.1093/jcag/gwaf042.287

https://doi.org/10.1093/jcag/gwaf042.288

https://doi.org/10.1093/jcag/gwaf042.289

https://doi.org/10.1093/jcag/gwaf042.290

https://doi.org/10.1093/jcag/gwaf042.291

https://doi.org/10.1093/jcag/gwaf042.292

https://doi.org/10.1093/jcag/gwaf042.293

https://doi.org/10.1093/jcag/gwaf042.294

https://doi.org/10.1093/jcag/gwaf042.295

https://doi.org/10.1093/jcag/gwaf042.296

https://doi.org/10.1093/jcag/gwaf042.297

https://doi.org/10.1093/jcag/gwaf042.298

https://doi.org/10.1093/jcag/gwaf042.299

https://doi.org/10.1093/jcag/gwaf042.300

https://doi.org/10.1093/jcag/gwaf042.301

https://doi.org/10.1093/jcag/gwaf042.302

https://doi.org/10.1093/jcag/gwaf042.303

https://doi.org/10.1093/jcag/gwaf042.304

https://doi.org/10.1093/jcag/gwaf042.305

https://doi.org/10.1093/jcag/gwaf042.306

https://doi.org/10.1093/jcag/gwaf042.307

https://doi.org/10.1093/jcag/gwaf042.308

https://doi.org/10.1093/jcag/gwaf042.309

https://doi.org/10.1093/jcag/gwaf042.310

https://doi.org/10.1093/jcag/gwaf042.311

https://doi.org/10.1093/jcag/gwaf042.312

https://doi.org/10.1093/jcag/gwaf042.313

https://doi.org/10.1093/jcag/gwaf042.314

https://doi.org/10.1093/jcag/gwaf042.315

https://doi.org/10.1093/jcag/gwaf042.316

https://doi.org/10.1093/jcag/gwaf042.317

https://doi.org/10.1093/jcag/gwaf042.318

https://doi.org/10.1093/jcag/gwaf042.319

https://doi.org/10.1093/jcag/gwaf042.320

https://doi.org/10.1093/jcag/gwaf042.321

https://doi.org/10.1093/jcag/gwaf042.322

https://doi.org/10.1093/jcag/gwaf042.323

https://doi.org/10.1093/jcag/gwaf042.324

https://doi.org/10.1093/jcag/gwaf042.325

https://doi.org/10.1093/jcag/gwaf042.326

https://doi.org/10.1093/jcag/gwaf042.327

https://doi.org/10.1093/jcag/gwaf042.328

https://doi.org/10.1093/jcag/gwaf042.329

https://doi.org/10.1093/jcag/gwaf042.330

https://doi.org/10.1093/jcag/gwaf042.331

https://doi.org/10.1093/jcag/gwaf042.332

https://doi.org/10.1093/jcag/gwaf042.333

https://doi.org/10.1093/jcag/gwaf042.334

https://doi.org/10.1093/jcag/gwaf042.335

https://doi.org/10.1093/jcag/gwaf042.336

https://doi.org/10.1093/jcag/gwaf042.337

